# Whole-genome resequencing reveals genetic diversity and selection signatures of Tongjiang and five goat breeds

**DOI:** 10.3389/fvets.2025.1559764

**Published:** 2025-05-27

**Authors:** Siyuan Zhan, Jing Luo, Ruiyang Li, Guangyao Li, Linxiang Li, Dandan Li, Tao Zhong, Linjie Wang, Jiazhong Guo, Jiaxue Cao, Hongping Zhang, Li Li

**Affiliations:** ^1^Key Laboratory of Livestock and Poultry Multi-Omics, Ministry of Agriculture and Rural Affairs, College of Animal Science and Technology, Sichuan Agricultural University, Chengdu, China; ^2^Farm Animal Genetic Resources Exploration and Innovation Key Laboratory of Sichuan Province, Sichuan Agricultural University, Chengdu, China; ^3^Bazhong Academy of Agriculture and Forestry Sciences, Bazhong, China

**Keywords:** whole-genome resequencing, Tongjiang goat, genetic diversity, population structure, selection signatures

## Abstract

One of the first livestock that humans domesticated was the goat. They give people access to vital supplies. However, the exploration and conservation of the genetic resources of goats are not comprehensive enough. Here, we generated whole-genome sequencing data for 35 samples from three populations (Tongjiang, Banjiao, and Chuandong white) and downloaded the genome sequence data of 30 individuals from three breeds (Jintang black, Chengdu grey, and Tibetan cashmere) to characterize the genomic variations of Tongjiang and other goat breeds. Among the six goat populations studied, the Tongjiang goat exhibits the highest levels of observed heterozygosity and runs of homozygosity, alongside the lowest inbreeding coefficient. The nucleotide diversity and linkage disequilibrium in this population were observed to be at intermediate levels. The population genetic structure analysis showed that significant differentiation of Tongjiang goats from other goat breeds, as evidenced by their distinct clustering and considerable genetic distance from other groups. Furthermore, based on selective sweeps, we identified positive selection genes in Tongjiang goats, which were probably related to important traits such as development (*BMP4*), reproduction (*HTR1E*), meat quality (*HDAC9*, *PITX2*, and *PDGFD*), and immune response (*IKBKB* and *DLG1*). Our findings indicate that Tongjiang goats possess elevated genomic diversity and exhibit minimal inbreeding. The unique genetic structure of Tongjiang goats can be partly attributed to the specific geographic conditions of the region. We have identified several selection signatures within the genomic regions of Tongjiang goats, which may play a role in their meat quality and environmental adaptation. All of our findings have the potential to provide an invaluable genomic resource for future genetic research and conservation efforts.

## 1 Background

Goats (*Capra hircus*) are one of the earliest domesticated livestock by humans. They have undergone domestication and extensive natural and artificial selection to adapt to various environments (1–3). They are widely distributed and diverse and provide people with basic living materials such as meat, milk, skin, and fiber (1). Domesticated goats spread around the world with human activity (4, 5). Globally varied and complex environments have contributed to the continual accumulation of genetic variation in the goat genome, resulting in a considerable amount of local goat genetic diversity. At present, Sichuan province has 17 goat breeds, including 11 local breeds (National Committee of Animal Genetic Resources, 2021).

The Tongjiang goat originated in Tongjiang County. It is worth noting that Tongjiang County is located northeast of Bazhong City, Sichuan Province. It is at the gap in Daba Mountain at the southern foot of the eastern section of Micang Mountain. The terrain is rough “three gorges and two valleys,” with steep mountains and deep ravines (6). Tongjiang goat is one of the precious livestock genetic resources in Sichuan Province. It has an extremely high economic value. Besides, Tongjiang goat skin was well-known in the international marketplace and was exported to Japan, Britain, Australia, and other nations as early as the 18th century. Tongjiang goat meat was highly valued for its nourishing and medicinal qualities (6).

Whole genome sequencing is increasingly accessible and cost-effective, making it a valuable tool in the study of genetic diversity. Research into the conservation and exploration of local goat genetic resources is expanding (7–13). For instance, Wang et al. (14) investigated the genetic diversity of eight goat breeds and identified genes associated with coat color patterns, body size, cashmere production, and adaptation to hypoxic conditions in high-altitude environments. Guo et al. (15) discovered abundant genetic diversity in six goat breeds, annotated with genes that may be related to goat coat color (*IRF4*, *EXOC2*, *RALY*, *EIF2S2*, and *KITLG*), high-altitude adaptation (*EPAS1*), growth (*LDB2*), and reproduction traits (*KHDRBS2*). Thereafter, they identified genes related to the Cashmere goat hair length (*FGF5*) and pigmentation (*KITLG*) (7). Gao et al. (11) identified a large number of variants in the genome of the Chongming goat and selected genes significantly associated with the nervous system, suggesting that the Chongming goat may be differentiated from other goat breeds in terms of the nervous system. However, investigations into genetic resources concerning genetic diversity and selection signatures of Chinese local goat breeds based on whole genome sequencing are limited.

**Figure 1:**
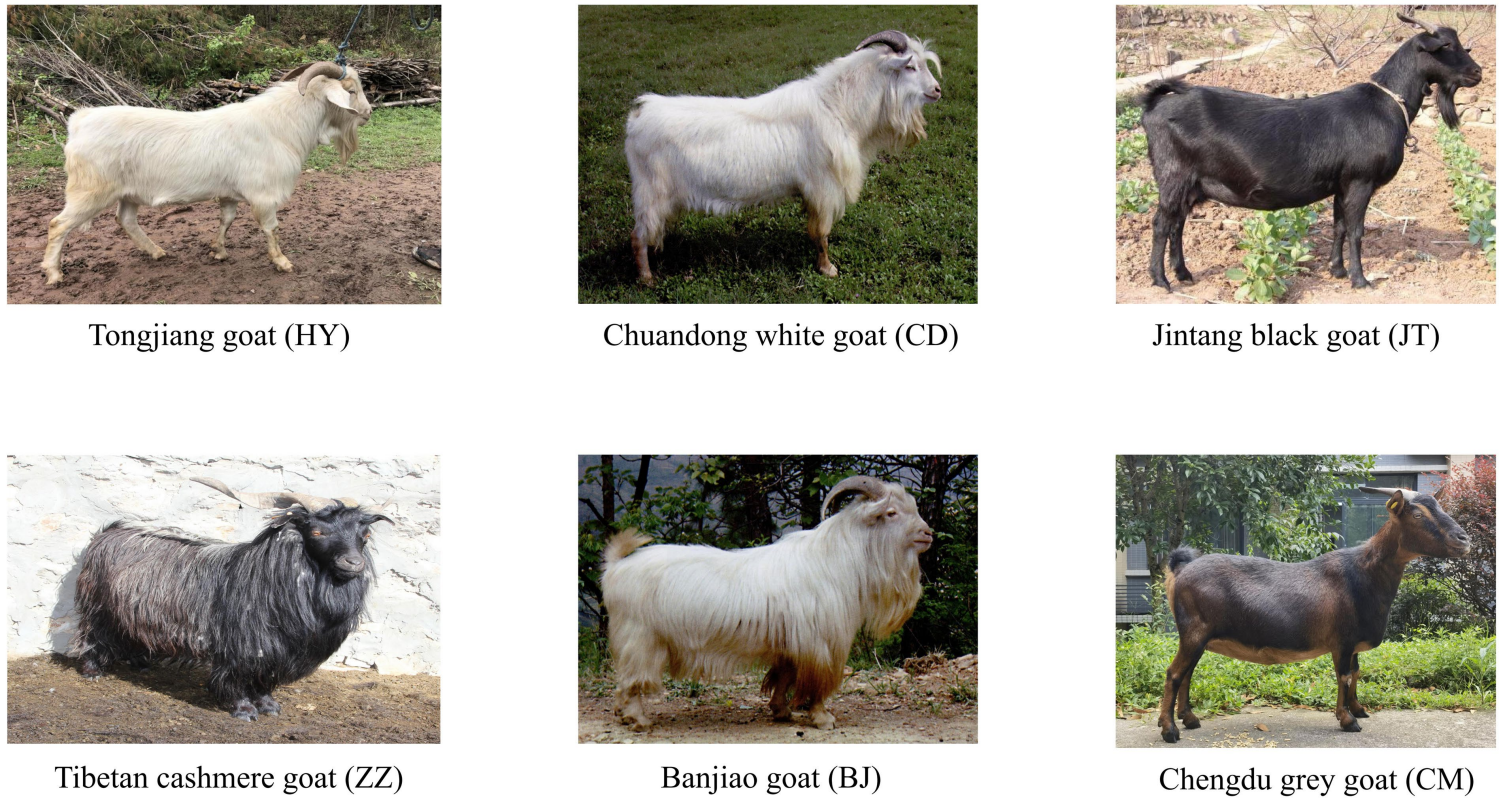
The six goat breeds included in this study (photographs were taken by Siyuan Zhan, Jiazhong Guo, and Hongping Zhang).

The Tongjiang goat is a crucial livestock genetic resource in Sichuan province, yet its utilization remains relatively low. To date, there have been no studies on the genome-wide genetic variation of Tongjiang goats. The Banjiao, Chuandong white, Jintang black, and Chengdu grey goats are among the typical goat breeds in Sichuan province. The Banjiao goat, primarily bred in Wanyuan County, Dazhou City, Sichuan Province, is a high-quality breed used for both meat and skin. It is characterized by its large body size, rapid growth rate, high meat yield, mild mutton flavor, large skin area, and superior skin quality. Moreover, it displays strong adaptability and disease resistance, making it an important genetic resource for developing herbivorous livestock and achieving the conversion of forage to meat in mountainous regions. The Chuandong white goat, inhabiting Dazhou City in Sichuan Province, is noted for its robust adaptability and strong reproductive capacity, despite its relatively small physique. The Chengdu grey goat, a superior indigenous goat breed in China, is mainly produced in Chengdu City, Sichuan Province. It is renowned for its good meat and milk production performance, high fertility, strong adaptability, and stable hereditability. The Jintang black goat, a specialty of Jintang County, Chengdu City, Sichuan Province, boasts a large body size, fast growth rate, high reproduction rate, strong adaptability, and disease resistance. Its body is covered with a glossy black coat. Additionally, Tibetan cashmere goat and goat breeds in Sichuan Province have similar distribution of high-quality genomic single nucleotide polymorphisms (SNPs), indicating a relatively close genetic relationship (7, 15). The Tibetan cashmere goat serves as an appropriate outgroup for comparative analysis with the Sichuan goat breeds. These goat populations exhibit clear economic and genetic significance, including traits such as meat, skin, and cashmere production. Historical records and genomic datasets are available to support robust comparisons. Furthermore, they represent diverse phenotypes in size, coat color, and adaptations to geographic environments (Figure 1).

A key objective of this study was to investigate the genetic relationship between Tongjiang goats and other major Sichuan breeds, as well as Tibetan cashmere goats. Given that Tongjiang goats are a precious but underutilized genetic resource with historical economic importance, understanding their genetic distinctiveness and relatedness to other breeds is crucial for conservation and breeding programs. Consequently, we have obtained whole genome sequencing data from a total of 65 individuals. Following that, the single nucleotide polymorphism (SNP) was identified by mapping to the reference genome of goats (ARS1). The Tongjiang goat and five other goat breeds were examined using SNPs to investigate their genetic diversity, population genetic structure, and selection signatures. This study aims to investigate the relationship between Tongjiang goats and the other four goat breeds in Sichuan Province, as well as Tibetan cashmere goats, to provide a reference for the protection and rational open utilization of genetic resources in goats.

## 2 Materials and methods

### 2.1 Sample collection and sequencing

The blood samples were randomly collected by jugular venipuncture from 15 Tongjiang goats (HY), 10 Banjiao goats (BJ), and 10 Chuandong white goats (CD) originating from Tongjiang, Wanyuan, and Dazhou Counties in Sichuan Province, China, respectively (Supplementary Table S1). The genomic DNA was extracted from the whole blood samples utilizing the blood DNA extraction kit (TIANamp Genomic DNA Kit, China) from the whole blood samples and stored at −20°C for future use. The integrity and purity of the extracted DNA were detected on 1% agarose gel electrophoresis. DNA concentration was measured by Qubit^®^ DNA Assay Kit in Qubit^®^ 3.0 Fluorometer (Invitrogen, United States). Subsequently, the qualified samples were sent for next-generation sequencing utilizing the Illumina platform and 150 bp paired-end reads to Novogene Bioinformatics Technology Co., Ltd. Additionally, resequencing data for 30 individuals comprising 10 Jintang black goats (JT), 10 Chengdu grey goats (CM), and 10 Tibetan cashmere goats (ZZ) were obtianed from public databases,^1^ with the accession number: PRJNA548681. A total of 65 whole genome resequencing data were used for subsequent analyses (Supplementary Table S1 and Figure 1).

### 2.2 Quality control and read mapping

Raw reads were quality-trimmed using Fastp (v0.19.7) to remove adapters, low-quality bases, and undetected bases. The error rate (%), quality scores (Q20 and Q30), and GC-content (%) of the clean reads after quality control were evaluated. Subsequently, the Burrows-Wheeler Aligner (v0.7.15-r1140) (16) with parameters (mem -t 4 -k 32 -M) was used to align the clean reads to the goat reference assembly ARS1 (GCF001704415.1). The mapped reads were sorted with SAMtools (v1.6) (17) software.

### 2.3 SNP calling

The SNP detection was performed on a cohort of 65 individuals using SAMtools mpileup, with the following parameter settings: -q 1, -C 50, -t SP, -t DP, -m 2, and -F 0.002. This was followed by variant calling using bcftools (version 1.21) (18). Concurrently, SNP filtering was executed employing SAMtools options dp3, -miss 0.1, and -maf 0.05. The filtering conditions include minor allele frequency less than 5%, missing data rate greater than 10%, and read coverage less than or equal to 3. SNPs were functionally annotated based on the goat reference assembly ARS1 using ANNOVAR (19) software.

### 2.4 Analysis of genetic diversity

The genomic nucleotide diversity serves as an indicator of the extent of genomic variation within a population. In this study, nucleotide diversity was assessed both at the population level and across the entire dataset using Stacks (v1.45) (20)^2^ and Variscan (v2.0) (21), respectively. Information about population genetic characteristics can be understood by calculating the runs of homozygosity (ROH). The ROH of 6 populations was statistically analyzed using PLINK (v1.07) (22) software. We separated the ROH length into four size classes to assess the ROH pattern of Tongjiang goats and other goat breeds: 0.5–1 Mb, 1–2 Mb, 2–5 Mb, and >5 Mb (7, 15). The parameters used were as follows: --homozyg-window-snp 50 --homozyg-snp 50 --homozyg-kb 500 --homozyg-density 50 --homozyg-gap 1,000 --homozyg-window-missing 5 --homozyg-window-threshold 0.05 --homozyg-window-het 03. The “-het” of the PLINK was used to obtain observed heterozygosity (Ho), expected heterozygosity (He), and inbreeding coefficient. The linkage disequilibrium (LD) decay for SNPs with different physical distances was calculated using PopLDdecay (v3.40) (23) software with default parameters.

### 2.5 Analysis of population structure and phylogenetic

In this study, GCTA software (v1.24.2) (24) software was employed to conduct principal component analysis (PCA), facilitating the computation of eigenvectors and eigenvalues. Subsequently, R software was utilized to generate the PCA plot. Additionally, the distance matrix was computed using TreeBest (v1.9.2)^3^ software, and the phylogenetic tree was constructed via the neighbor-joining (NJ) method, with a bootstrap value set at 1,000. The admixture analysis of the six goat populations was performed using the Admixture (v1.23) (25) software, following the preparation of the Ped file, an input file for PLINK. Utilizing SMC++ (v1.15.0),^4^ the effective population size of Tongjiang goats was estimated with the parameter configuration of -c 10 for chromosome segmentation, -r 1.5 × 10^−8^ for the recombination rate, -g 6 for the generation time, -N 100 for the initial effective population size, and -m smc_prime for the demographic model. We employed TreeMix (v1.13) (26)^5^ software to investigate population differentiation and gene flow.

### 2.6 Detection of selection signatures of Tongjiang goat

Identifying regions of selective elimination through the analysis of nucleotide diversity (θπ) and the population fixation index (F_ST_) has proven to be a highly effective approach (27). Strong selection signals are frequently obtained when mining functional regions directly related to the living environment. In this study, the F_ST_ and θπ were calculated to identify selection regions in Tongjiang goat genome, utilizing a sliding window approach with a window size of 20 kb and a step size of 10 kb, implemented via VCFtools (v0.1.14) (28) software. Furthermore, Tajima’s D test was employed to further detect selection (29). The parameters specified for the calculation of Tajima’s D using VCFtools included “--vcf 20F.vcf –Tajima’s D 500000 --out Tajima's D.”

### 2.7 Function enrichment analysis

Perform functional annotation utilizing the Gene Ontology (GO) and Kyoto Encyclopedia of Genes and Genomes (KEGG) databases, and enhance gene function characterization in the selected regions by applying filters based on *p*-values. For GO and KEGG functional enrichment analysis, we employed KOBAS-i^6^ (30) and DAVID^7^ (31), respectively.

## 3 Results

### 3.1 Genome resequencing

This study involved the whole genome resequencing of 35 individuals, producing 1325.77G of raw data and 1324.08G of clean data after filtering. Q20 greater than 96.87%, Q30 more than 89.06%, and GC content bigger than 41.99% were present in all sequencing samples (Supplementary Table S1). In addition, a total of 667.86G of raw data from 30 individuals was downloaded from the public database. A total of 65 resequencing data were selected for analysis. In the completely aligned reference genome reads set, the average sequencing depth was estimated to be 10.13×, and the average mapping rate was 99.61%. The above results indicate that high-quality sequences were obtained in this study and can be used for further analysis (Supplementary Table S2).

### 3.2 SNP calling and annotation

In our analysis of whole genome data from 65 samples, we identified a total of 28,737,083 SNPs. Following rigorous filtering procedures, 13,611,773 SNPs were retained for further study. Among the samples, the Tongjiang goat exhibited the highest number of SNPs, totaling 11,795,740, while the Tibetan goat displayed the lowest, with 11,177,956 SNPs. Annotation of the SNPs revealed that 27.92% were located within intronic regions, 1.05% within exon regions, with 0.38% being synonymous and 0.26% non-synonymous. The ratio between transition and transversion was calculated to be 2.42, as detailed in Supplementary Table S3.

### 3.3 Genetic diversity of six goat populations

**Figure 2:**
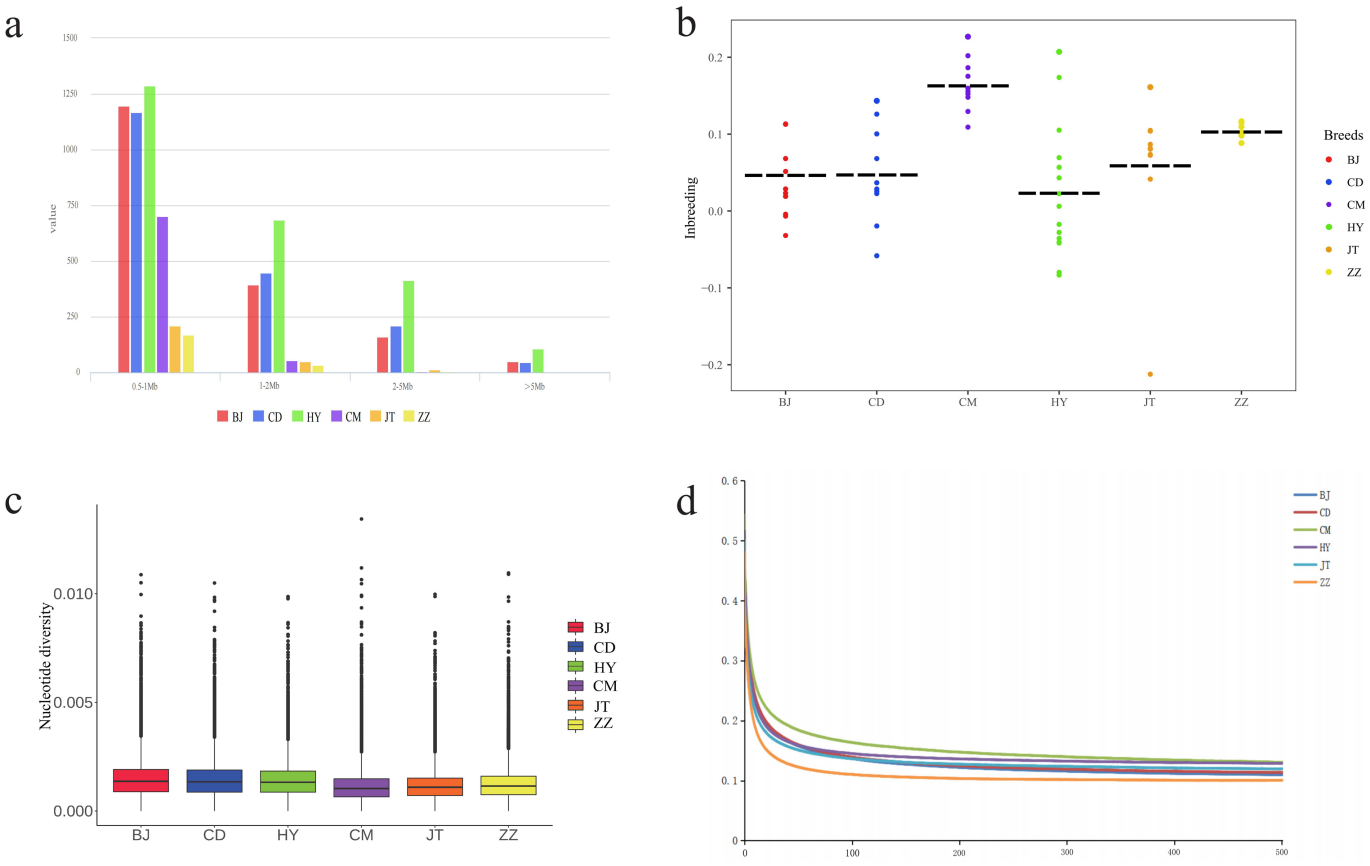
Genomic genetic diversity statistics of 6 goat populations. **(a)** The distribution of total number of ROH across chromosomes. **(b)** Statistics on inbreeding coefficients of each goat population. **(c)** Genome-wide distribution of nucleotide diversity from each population. **(d)** Genome-wide average LD decay estimated in each population.

We evaluated the ROH, Pi, heterozygosity, LD, and inbreeding coefficient of six goat populations (Figure 2 and Supplementary Tables S4–S7. The ROH of each breed was divided by length (0.5–1 Mb, 1–2 Mb, 2–5 Mb and >5 Mb). The results showed that the majority of ROH was found to be between 0.5 and 1 Mb in all goat populations. Surprisingly, Tongjiang goat had the most ROH in each segment (Figure 2a and Supplementary Table S4). The nucleotide diversity of Tongjiang goat (0.00128) was close to that of Banjiao goat (0.00133) and Chuandong white goat (0.00131), both of which had higher genetic diversity, while that of Chengdu grey goat (0.00100) was the lowest (Figure 2c and Supplementary Table S1). At a distance of 100 kb, the Chengdu grey goat exhibited the highest average LD level across the genome. The LD levels of the Tongjiang goat, Jintang goat, and Chuandong white goat were similar. Notably, the Tibetan cashmere goat displayed the lowest LD level and the fastest rate of LD decay (Figure 2d and Supplementary Table S6).

The Ho was highest in the Tongjiang goat at 0.2931, whereas the Chengdu grey goat had the lowest Ho at 0.2507. The He of six goat populations was remarkably similar (Supplementary Table S5). Conversely, Chengdu grey goat had the highest inbreeding coefficient (0.1576), while Tongjiang goat had the lowest inbreeding coefficient (0.0061) (Figure 2b and Supplementary Table S7).

### 3.4 Population structure of six goat populations

**Figure 3:**
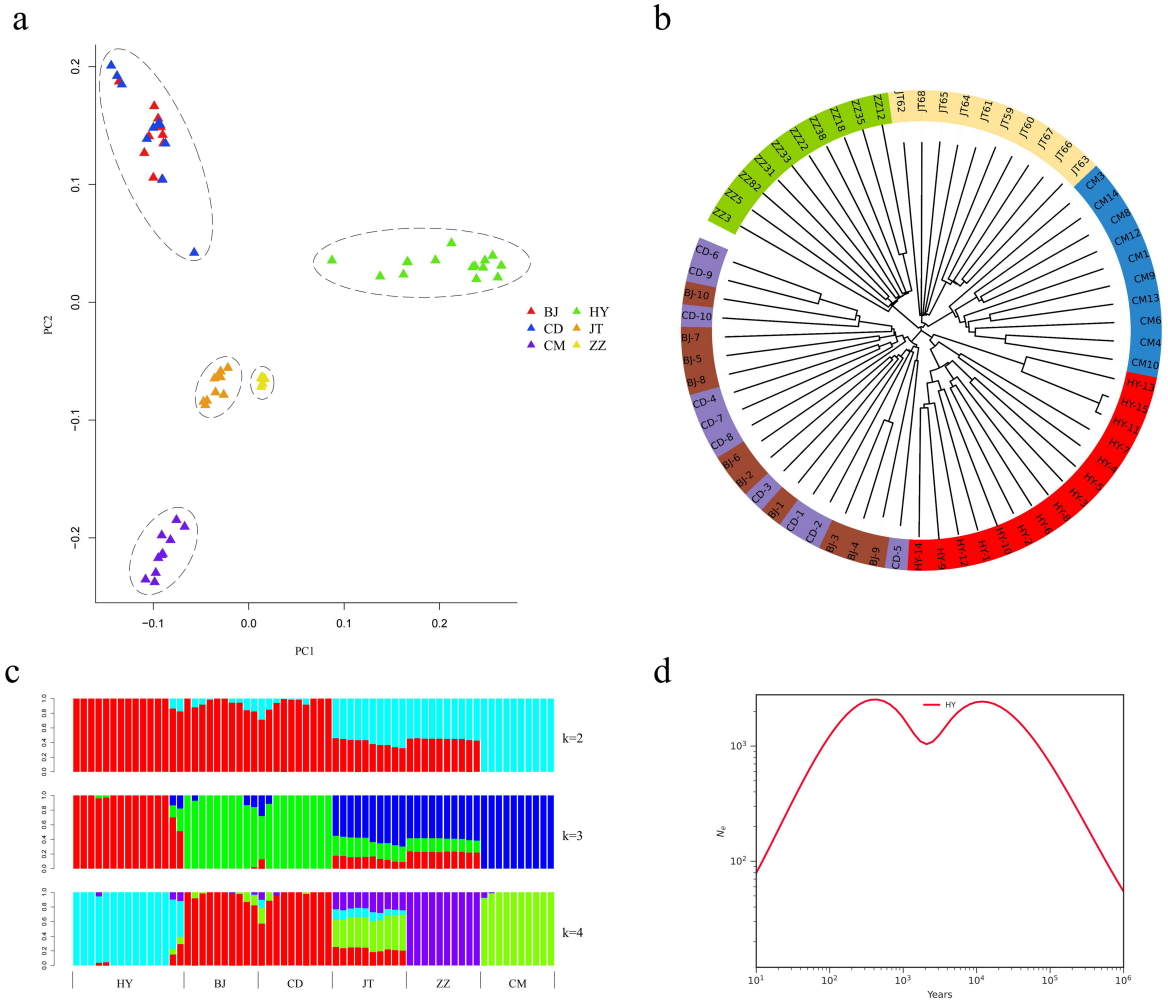
Population genetic structure and relationships of six goat populations. **(a)** Principal component analysis of six goat populations (65 individuals). **(b)** Neighbor-joining tree of the relationships between the six goat populations. **(c)** The clustering of goat populations using ADMIXTURE with *K* = 2 and *K* = 4. **(d)** Effective population size of the SMC++ population history of Tongjiang goats.

**Figure 4:**
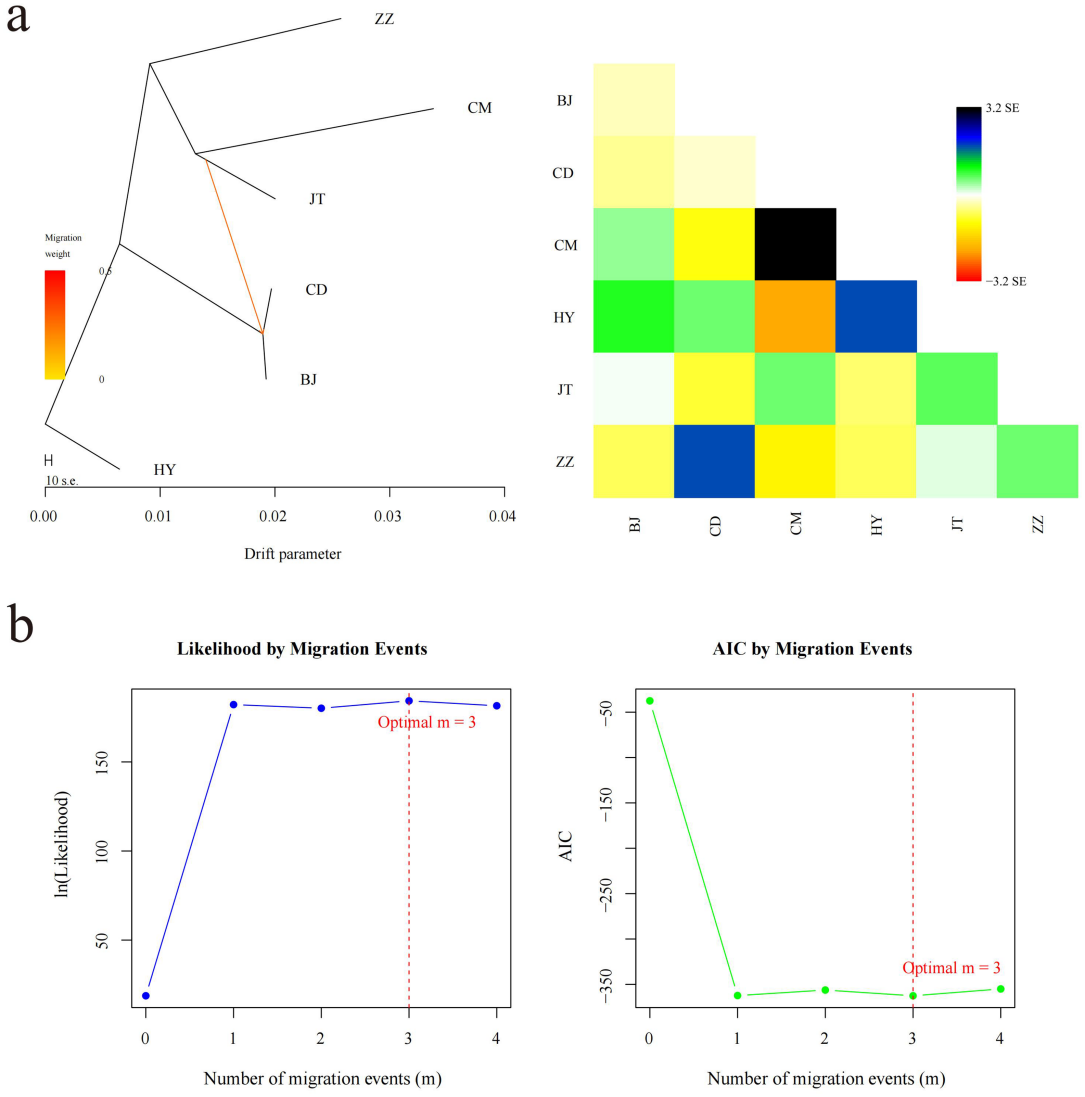
Inference of gene flow among six goat populations. **(a)** The evolutionary tree and heat map of one gene flow among six goat populations. The scale below shows the 10 times average standard deviation of the elements in the sample covariance matrix. **(b)** Optimal number of migration events in gene flow analysis. The change in the likelihood function with the number of migration events is shown on the left side. Change in Akaike information criterion (AIC) with the number of migration events on the right side.

In this study, we examined the phylogenetic relationships among 65 samples from six goat populations utilizing 29 pairs of autosomal SNPs. The NJ tree analysis revealed that the 65 individuals were clustered into five distinct branches. Notably, each population of Tongjiang goat, Tibetan cashmere goat, Chengdu grey goat, and Jintang black goat constituted separate clades, while the Chuandong white goat and Banjiao goat populations formed a separate clade (Figure 3a). The PCA corroborated these findings, displaying five clusters that aligned with the NJ tree results. Specifically, the Tongjiang goats were identified as a distinct cluster, exhibiting characteristics markedly divergent from those of the other goat breeds (Figure 3b). Chuandong white goats and Banjiao goats were grouped together and close to each other. This outcome is likewise supported by the maximum likelihood tree in the gene flow study (Figure 4).

We estimated the ancestral populations of 65 goat samples using clustering models. According to the admixture estimates from *K* = 2 to *K* = 4, goat populations gradually separated from one another. In the case of *K* = 2, the 65 goats were divided into two major genetic groups, while goats have been reported in the literature to have undergone two population expansions in Asia (32), which may correspond to two different genetic origins or expansion events. When *K* = 3, Tongjiang goat was distinctively separated. Further differentiation at *K* = 4 resulted in the separation of the Chengdu grey goat, while Jintang black goat exhibited mixed phenomena (Figure 3c).

Utilizing SMC++, an analysis of the effective population size history of the Tongjiang goat revealed a dynamic pattern characterized by a rapid increase in population size during the early stages, followed by a gradual decline after reaching a peak. Subsequently, the population size ascended to a new zenith before trending downwards once more. This pattern likely reflects the various environmental and genetic pressures encountered by the Tongjiang goats throughout their evolutionary process (Figure 3d).

We conducted a gene flow analysis to investigate the genetic exchange between populations utilizing TreeMix software (Figure 4). Under the assumption of a single migration event, the analysis indicates a gene flow direction from the Jintang black goat to both the Chuandong white goat and the Banjiao goat, with a relatively high migration weight (Figure 4a). While OptM analysis suggested *m* = 3 as the optimal number of migration events, we ultimately selected *m* = 1 for model parsimony based on likelihood improvement from *m* = 1 to *m* = 3 was marginal compared to the substantial gain from *m* = 0 to *m* = 1 (Figure 4b). Notably, the Tongjiang goat consistently remained distinct from other goat populations in the maximum likelihood tree (Figure 4).

### 3.5 The selection signatures analysis of Tongjiang goats

**Figure 5:**
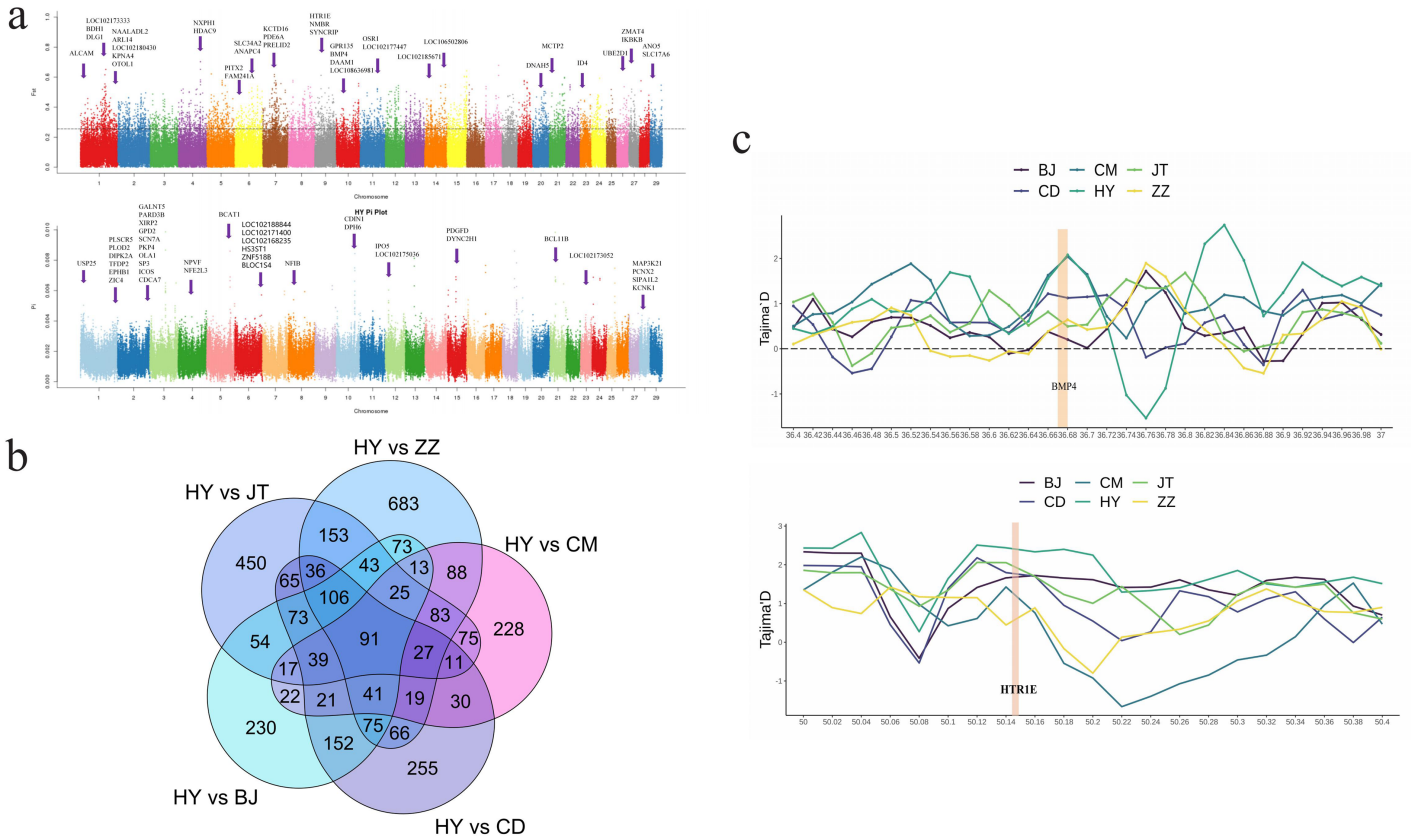
Analysis of the signatures of positive selection in the genome of Tongjiang goat. **(a)** Manhattan plot based on θπ and F_ST_ selection signal analysis in Tongjiang goat. **(b)** Venn diagram of Tongjiang goat and other goat breeds using θπ and F_ST_ selection signal analysis. **(c)** The Tajima’s D at the *BMP4* and *HTR1E* gene region.

The analysis of nucleotide diversity (θπ) and population fixation index (F_ST_) were employed to investigate the selected genomic regions in Tongjiang goat and five other goat breeds. Selection signature analysis identified 1,622 putative selection genes within the top 5% between Tongjiang goats and Tibetan cashmere goats, 830 genes between Tongjiang goats and Chengdu grey goats, 1,075 genes between Tongjiang goats and Banjiao goats, 1,107 genes between Tongjiang goats and Chuandong white goats, and 1,348 genes between Tongjiang goats and Jintang black goats (Supplementary Table S8 and Figure 5a; Supplementary Figure S1). A total of 91 genes were obtained through overlapping selection genes (Supplementary Table S9 and Figure 5b).

### 3.6 Functional enrichment analysis of overlapping selection genes

**Figure 6:**
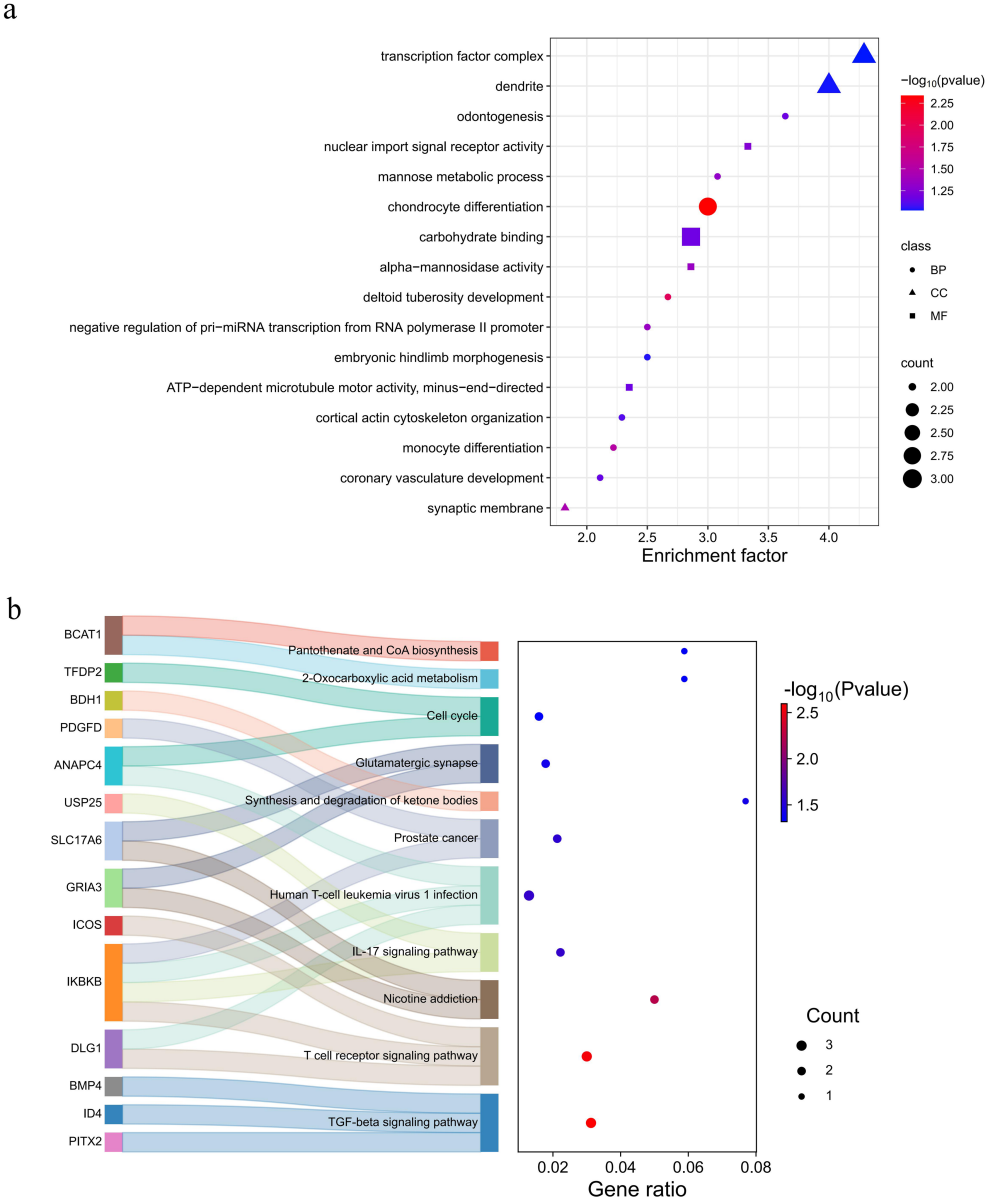
Enrichment analysis of 91 selected genes in Tongjiang goats. **(a)** The GO enrichment analysis selected genes in Tongjiang goats. **(b)** The KEGG enrichment analysis selected genes in Tongjiang goats.

Functional enrichment analysis was conducted using KEGG and GO databases for overlapping genes. There were seven significant (*p* < 0.05) GO terms were enriched, encompassing biological processes such as chondrocyte differentiation, deltoid tuberosity development, monocyte differentiation, synaptic membrane, negative regulation of pri-miRNA transcription from RNA polymerase II promoter, alpha-mannosidase activity, and mannose metabolic process (Figure 6a and Supplementary Table S10). Notably, chondrocyte differentiation exhibited a highly significant enrichment (*p* < 0.01) enriched, involving three genes (*BMP4*, *NFIB*, and *OSR1*). These genes are implicated in development processes (33), inflammatory responses (34), diseases (35, 36), and cancer (37, 38). This phenomenon may be attributed to the adaptability of Tongjiang goats to complex environments. The KEGG pathway revealed 11 pathways that were statistically significant (*p* < 0.05) in Tongjiang goats. Among these, the TGF-beta signaling pathway, T cell receptor signaling pathway, and Nicotine addiction pathway were highly enriched (*p* < 0.01) enriched involving relevant genes (*PITX2*, *ID4*, *BMP4*, *DLG1*, *IKBKB*, *ICOS*, *GRIA3*, and *SLC17A6*) (Figure 6b and Supplementary Table S11). Furthermore, the most pronounced results from the Tajima’s D analysis in Tongjiang goats provided substantial evidence of a positive selection region, encompassing the *BMP4* and *HTR1E* genes (Supplementary Table S12).

## 4 Discussion

To elucidate the differences in genetic variation between Tongjiang goats and five other goat breeds, we conducted a comparative analysis focusing on genetic diversity, population structure, phylogenetic relationships, and selection characteristics. Our findings revealed that the length and distribution density of ROH in Tongjiang goat were greater than those observed in the other goat breeds. Conversely, the inbreeding coefficient of Tongjiang goats was lower, suggesting a reduced level of inbreeding within this population. This phenomenon may be attributed to selective pressures that favor certain genotypes, enhancing their adaptability to specific environmental conditions (39). The nucleotide diversity of Banjiao goat, Chuandong white goat, and Tongjiang goat was relatively high, which were 0.001, 0.10021, and 0.0031, respectively (Supplementary Table S4). The LD decay patterns of Banjiao goat, Chuandong white goat, and Tongjiang goat were similar, confirming the high genetic diversity. This may be related to their similar living environment. Banjiao goat, Chuandong white goat, and Tongjiang goat all live in the ridge valley area of eastern Sichuan Province.

The analysis of the population genetic structure indicated that the Tongjiang goat was independently categorized into a distinct cluster, exhibiting a relatively large genetic divergence from other goat breeds. In contrast, the Banjiao goat and Chuandong white goat were grouped into a single cluster, demonstrating closer genetic proximity (Figure 3). These findings align with the geographical distribution of these breeds, as the primary production regions for Banjiao and Chuandong white goats are in close geographical proximity. Nevertheless, the primary production areas of Tongjiang goats and Banjiao goats were geographically separated by the Tongjiang River, and similarly, the primary production areas of Tongjiang goats and Chuandong white goats were demarcated by the presence of the Tongjiang River. The gene flow analysis revealed significant genetic exchange among Jintang black goats, Chuandong white goats, and Banjiao goats. Jiang et al. (40) also speculated, through the analysis of amplified fragment length polymorphism (AFLP) markers, that there was gene flow between Chuandong white goat and Banjiao goat. The Jintang black goats may have had a considerable impact on the genetic composition of both Chuandong white goats and Banjiao goats. Notably, Tongjiang goats consistently exhibited genetic distinctiveness from other goat populations in the maximum likelihood tree, implying a unique genetic background or a divergent evolutionary path. This may reflect the geographical isolation or specific selective pressures experienced by Tongjiang goats, resulting in their unique genetic profile.

Tongjiang goats represent a valuable asset in the field of animal husbandry due to their exceptional traits, which encompass fresh and tender meat, a dense and uniform cortex, and superior adaptability to environmental conditions (6). Consequently, we performed a selection feature analysis on Tongjiang goats, utilizing five other goat breeds as the reference population. Through the analysis of θπ and F_ST_ selection signals, the overlapping candidate gene (*BMP4*) emerged as significant in both development and disease. The *BMP4* gene exhibits pleiotropic effects and plays essential roles in many developmental processes, including myogenic differentiation (41), neurogenesis (42), vascular development, angiogenesis (43), and osteogenesis (44). Diseases associated with BMP4 include microphthalmia (45) and orofacial cleft (46). Additionally, The *BMP4* gene was associated with the growth (47) and reproductive performance of goats (48, 49). Furthermore, prior research has demonstrated that miRNA-378 plays a crucial role in promoting myoblast differentiation by targeting *BMP4* (41). The expression of mRNA transcribed from the *BMP4* gene varied in skin tissue from anagen to telogen phases (50). The literature indicates that BMP4 plays a crucial role in significantly enhancing post-weaning daily weight gain, as well as modulating body weight indices and contributing to the development of skeletal muscle in lambs (51). This variation partially accounts for the distinct skin and meat characteristics observed in Tongjiang goats. Subsequently, the verification analysis was performed on the gene region to prevent false positives. The highest Tajima’D analysis values in Tongjiang goats strongly supported the inclusion of the *BMP4* gene in the positive selection region.

A study has identified a correlation between the *HDAC9* gene and the development of muscle structure in sheep (52). Additionally, research has demonstrated that *PIXT2* plays a significant role in regulating muscle growth and is closely associated with myogenesis (53). Zhang et al. (54) identified *PDGFD* as a potential gene linked to fat deposition. Notably, Dianzhong cattle exhibit a high propensity for intramuscular fat accumulation, contributing to the production of marbled beef. Therefore, we speculate that these genes may be related to goat meat quality traits. Tongjiang goats may enhance meat quality through the regulation of myogenesis and fat deposition. In addition, two genes (*DLG1* and *IKBKB*) associated with immunity displayed signals of positive selection in Tongjiang goats. *DLG1* was important for human dendritic cell 83 (CD83) up-regulation and IL-6 production upon maturation, and its proteins are essential for sufficient IL-12 production after maturation (55, 56). This could affect innate and adaptive immune responses. The *IKBKB* gene was an inhibitor of NF-kappaB transcription factors, while transcription factors of the NF-kappaB protein family were a key regulatory factor in immunity, inflammation, and cancer (57, 58). The *DLG1* and *IKBKB* genes are significant in facilitating the environmental adaptability of Tongjiang goats. Hernandez-Montiel et al. (59) believed that the *HTR1E* gene was a candidate gene related to sheep litter size based on genome-wide association analysis. Soon afterward Tao et al. (60) study found that *HTR1E* was related to the total number of lambs born in sheep. The positive selection region including the *HTR1E* gene was intensively confirmed by the highest Tajima’D values in Tongjiang goats (Figure 5c). Based on our findings, we speculate that the *HTR1E* genes may contribute to reproductive performance in Tongjiang goats.

We performed GO and KEGG enrichment analyses of overlapped candidate genes. The GO enrichment analysis revealed significant terms associated with developmental processes, such as chondrocyte differentiation, deltoid tuberosity development, and monocyte differentiation. Additionally, the KEGG pathway analysis identified significant enrichment in the TGF-beta signaling pathway and T cell receptor signaling pathway (Figure 6). Notably, TGF-beta signaling pathway is an important cell signaling pathway that plays a key role in development (61), immune regulation (62), and disease occurrence (63). The T cell receptor signaling pathway plays a crucial role in the activation of T cells, which are essential for the regulation of immune responses (64). These results suggest that Tongjiang goats may have enhanced immunological and developmental capacity, though further experimental validation is required to confirm these findings. In the future, the sample size could be expanded and more experimental validation could be performed to explore the association of candidate genes with important phenotypic traits in more depth.

## 5 Conclusion

This study elucidates the unique genetic characteristics of Tongjiang goats through the analysis of whole genome resequencing data. Geographic isolation has contributed to the particular genetic diversity and population structure of Tongjiang goats, providing reference value for the assessment of goat genetic resources and reasonable breeding of Tongjiang goats. Moreover, we identified candidate genes associated with meat quality traits, goatskin quality, environmental adaptability, disease resistance, and immune response in the Tongjiang goat. It is essential that future research endeavors include comparative experimental studies to evaluate the phenotypic expression of these genes across various goat breeds. This study holds significant reference implications for future research into the genomic characteristics of other important local goat resources globally.

## Data Availability

The datasets generated for this study are publicly available in China National Center for Bioinformation repository, https://ngdc.cncb.ac.cn under accession link: [https://ngdc.cncb.ac.cn/search/specific?db=biosample&q=PRJCA022316. All commands and scripts used in this study have been uploaded as Supplementary material.

## Footnotes

^1^https://www.ncbi.nlm.nih.gov/sra/

^2^https://catchenlab.life.illinois.edu/stacks/

^3^http://treesoft.sourceforge.net/treebest.shtml

^4^https://github.com/popgenmethods/smcpp/tree/v1.15.0

^5^https://bitbucket.org/nygcresearch/treemix/downloads/

^6^http://kobas.cbi.pku.edu.cn

^7^https://david.ncifcrf.gov

## Supplementary material

SUPPLEMENTARY FIGURE S1 The θπ and Fst selection signal analysis between Tongjiang goat and other goat breeds. **(a)** The θπ and *F*_st_ selection signal analysis between Tongjiang goat and Banjiao goat. **(b)** The θπ and *F*_st_ selection signal analysis between Tongjiang goat and Chuandong white goat. **(c)** The θπ and *F*_st_ selection signal analysis between Tongjiang goat and Jingtang black goat. **(d)** The θπ and *F*_st_ selection signal analysis between Tongjiang goat and Chengdu grey goat. **(e)** The θπ and *F*_st_ selection signal analysis between Tongjiang goat and Tibetan cashmere goat.
