# Supplementary figures and images for: Whole-genome resequencing reveals genetic diversity and selection signatures of Tongjiang and five goat breeds
Source: Front Vet Sci. 2025 May 27;12:1559764. doi: 10.3389/fvets.2025.1559764 (PMC12150802; doi:10.3389/fvets.2025.1559764)

a

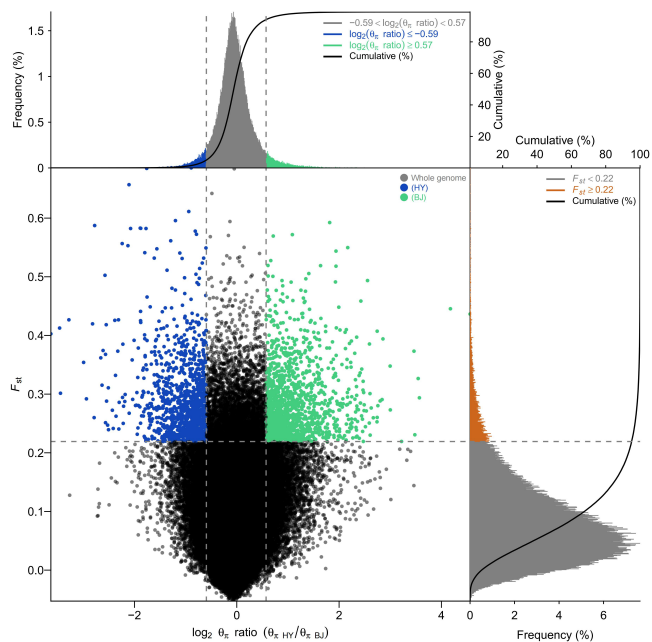

b

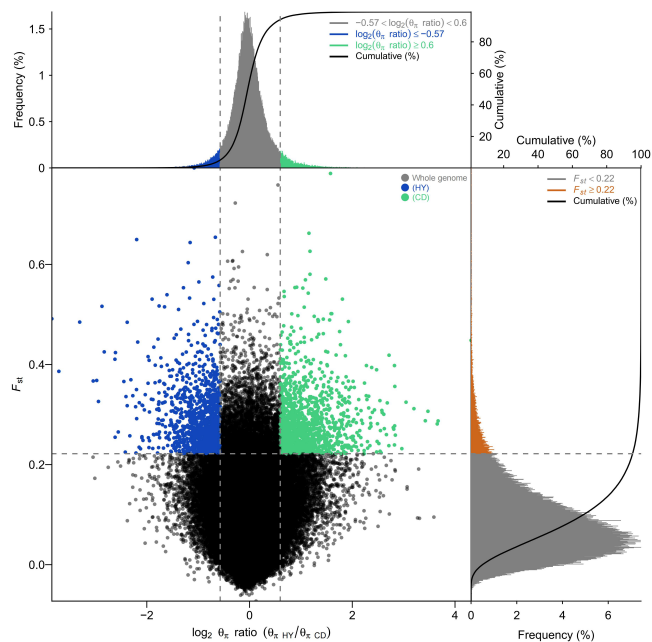

c

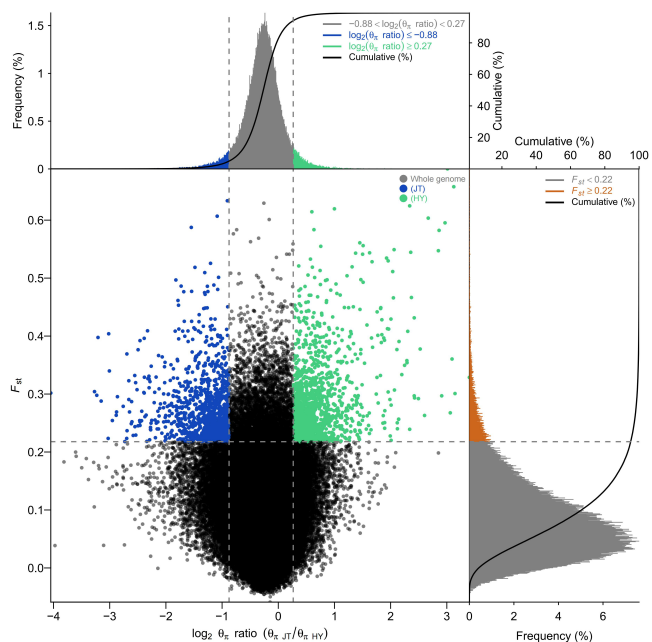

d

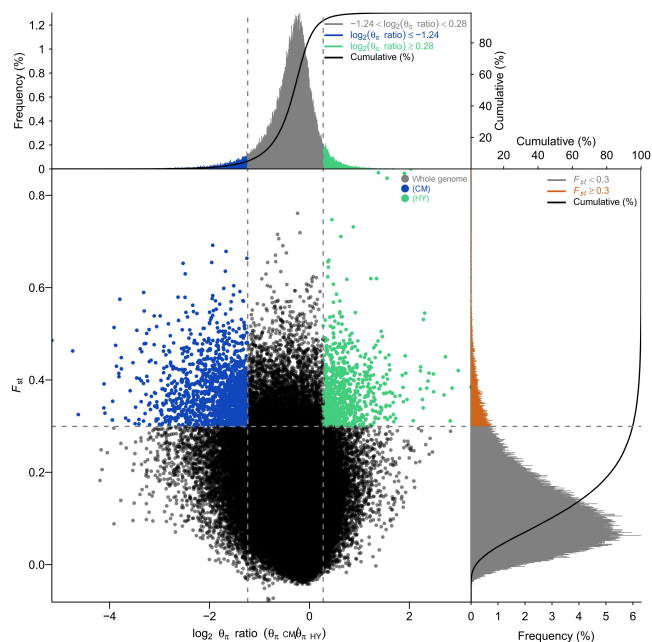

e

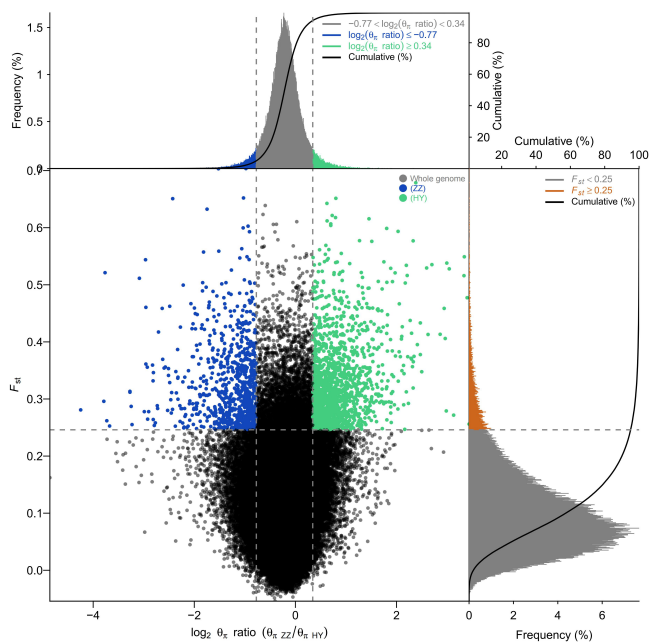

Supplement: SUPPLEMENTARY FIGURE S1 — The θπ and Fst selection signal analysis between Tongjiang goat and other goat breeds. (a) The θπ and Fst selection signal analysis between Tongjiang goat and Banjiao goat. (b) The θπ and Fst selection signal analysis between Tongjiang goat and Chuandong white goat. (c) The θπ and Fst selection signal analysis between Tongjiang goat and Jingtang black goat. (d) The θπ and Fst selection signal analysis between Tongjiang goat and Chengdu grey goat. (e) The θπ and Fst selection signal analysis between Tongjiang goat and Tibetan cashmere goat. [file Image_1.pdf]
